# Dewetting-Induced Hierarchical Self-Assembly of Block Copolymers Templated by Colloidal Crystals

**DOI:** 10.3390/polym15040897

**Published:** 2023-02-11

**Authors:** Dong Hwan Kim, Hong Gu Kwon, Hong Kyoon Choi

**Affiliations:** Division of Advanced Materials Engineering, Kongju National University, Cheonan 31080, Republic of Korea

**Keywords:** directed self-assembly, block copolymers, colloidal crystal, self-assembly, dewetting, surface-directed dewetting

## Abstract

Recent advances in high-performance flexible electronic devices have increased the demand for more diverse and complex nanofabrication methods; high-resolution, high-efficiency, and low-cost patterning strategies for next-generation devices are therefore required. In this study, we demonstrate the formation of dewetting-induced hierarchical patterns using two self-assembled materials: block copolymers (BCPs) and colloidal crystals. The combination of the two self-assembly methods successfully generates multiscale hierarchical patterns because the length scales of the periodic colloidal crystal structures are suitable for templating the BCP patterns. Various concentric ring patterns were observed on the templated BCP films, and a free energy model of the polymer chain was applied to explain the formation of these patterns relative to the template width. Frequently occurring spiral-defective features were also examined and found to be promoted by Y-junction defects.

## 1. Introduction

Block copolymers (BCPs) have been extensively studied for decades, owing to their inherent ability to produce highly ordered two-/three-dimensional (2D/3D) nanostructures. BCPs have many applications in diverse fields, including electronics [1,2,3], photonics [4,5,6,7], plasmonics [8,9], filtration [10,11,12], and energy devices [13,14,15,16]. In particular, with the development of various methodologies for controllable BCP patterning, such as directed self-assembly (DSA), BCPs have attracted considerable attention as candidates for next-generation lithography. The DSA method guides the BCP patterning process with graphoepitaxial or chemoepitaxial templates which are typically fabricated using conventional lithography techniques, such as photolithography and e-beam lithography [17,18,19,20,21].

Similar to BCPs, colloidal crystals obtained via the self-assembly of colloidal particles can be used in bottom-up nanofabrication for generating regular patterns with high efficiency and low cost [22,23,24,25,26,27]. Significantly, the length scales of periodic colloidal crystals, which range from hundreds of nanometers to several micrometers, are highly suitable for their application as guiding templates for the patterning of BCPs. Despite this compatibility, there have been relatively few reports on the control of BCP patterns using colloidal crystal templates; for example, Brassat et al. and Jung et al. previously reported the fabrication of BCP patterns inside topographic templates prepared via metal deposition through a lithographic mask composed of colloidal crystals [28,29,30].

The dewetting of thin films, which is a separation phenomenon induced by incompatible interfacial energies between the surface and film, is another useful method for micro/nanoscale separation or patterning of various materials, including metals, nanoparticles, and polymers [31,32,33,34,35]. In particular, the dewetting of BCP thin films has been shown to produce micro/nanoscale hierarchical patterns [36,37]. To control dewetting more precisely, a surface-directed dewetting method using a guiding template, fabricated by either chemical or topographic patterning, has been proposed [38,39,40,41,42]. Among these two methods, chemical patterning, which induces dewetting based on the differences in the surface energy across the patterned area, is the simpler and more versatile method.

In this study, we demonstrate the formation of dewetting-induced hierarchical patterns by combining two self-assembled materials: block copolymers and colloidal crystals. Here, a chemically patterned template was used to direct the dewetting of the BCP film. Various concentric ring patterns were formed, the dimensionality and number of which depended on the diameter or spacing of the circular guiding template. A free energy model of the polymer chains was then applied to explain the behavior of BCP pattern formation through the guiding template. In addition, the generation of frequently occurring spiral-defective features was examined in detail.

## 2. Materials and Methods

### 2.1. Preparation of Colloidal Particles and Multilayered Colloidal Crystals

Polystyrene (PS) particles with diameters of 250 and 430 nm were synthesized using emulsifier-free emulsion polymerization [43]. To prepare the 430 nm PS colloidal particles, deionized water (450 mL) was first poured into a reactor and maintained at 70 °C while stirring at 350 rpm. Thereafter, sodium styrene sulfonate (0.06 g), comonomer, and sodium hydrogen carbonate (0.25 g), a buffer, were added to the reactor. After 10 min, the styrene monomer (50 g) was added to the solution. After 1 h, potassium persulfate (0.25 g) was added as an initiator. Polymerization was performed for 18 h in a nitrogen atmosphere. The same procedure was used to prepare 250 nm PS colloidal particles; however, the content of sodium styrene sulfonate was modified to 0.75 g for these reactions.

Next, 1100 nm PS particles were synthesized using dispersion polymerization [44]. First, 0.01 g of poly(vinylpyrrolidone) (PVP) (Mw = 10,000, Sigma-Aldrich, St. Louis, MO, USA) was dissolved in 25 mL of ethanol (99.5 %, Samchun chemicals, Seoul, South Korea). Subsequently, 2 g of styrene monomer (Sigma-Aldrich) was added, and the solution was stirred at 350 rpm at 70 °C to ensure complete mixing. Thereafter, 3 mL of 5 mM aqueous ammonium persulfate (APS, Sigma-Aldrich) was added to the solution. Polymerization was performed for 18 h with sufficient stirring.

To fabricate the multilayered colloidal crystals, a 0.1 wt.% PS colloidal suspension in 40 mL deionized water was prepared with 0.001 wt.% PVP (Mw = 55,000, Sigma-Aldrich). A glass slide was cleaned with ethanol and treated with O_2_ plasma (2 min, 50 W, Tergeo plasma cleaner, PIE Scientific, Union City, CA, USA) to render the surface hydrophilic. The glass slide was then coated by dipping it vertically into the PS colloidal suspension and placed in an oven at 70 °C overnight [45].

### 2.2. Preparation of the Guiding Template

A PS brush layer was applied to a silicon substrate by spin coating a 1% hydroxyl-terminated PS (Mw = 11 kg mol^−1^, Polymer Source Inc., Dorval, QC, Canada) solution in toluene (99.5 %, Daejung chemicals & metals, Gyeonggi-do, South Korea), followed by baking at 170 °C for 12 h and rinsing. A non-close-packed hemispherical colloidal crystal monolayer was then deposited on the PS brush substrate by transferring the surface layer of a multilayered colloidal crystal via thermal transfer printing [46]. For the transfer, the prepared multilayered colloidal crystal was exposed to an O_2_ plasma (2–4 min, 90 W. Thereafter, a flat PDMS pad was placed on the surface-etched crystal with an applied pressure of approximately 100 kPa. The surface colloidal layer was subsequently peeled away by lifting the PDMS pad from the colloidal crystal. Afterward, the PDMS pad was placed on the heated PS brush substrate (which was maintained above the glass transition temperature (T_g_) of PS; ~115 °C), and a small pressure was applied for conformal contact. A non-close-packed hemispherical colloidal crystal monolayer on the PS-brushed substrate was obtained by peeling the PDMS pad from the substrate.

The exposed sections of the PS brush layer were then removed by O_2_ plasma etching (2 min, 90 W). Afterward, a fluorinated self-assembled monolayer (F-SAM) was deposited on the exposed areas of the substrate by dipping the sample in a 0.5 wt.% solution of heptadefluoro-1,1,2,2-tetrahydrodecyl trichlorosilane (HDFS, Sigma-Aldrich) in n-hexane (95 %, Duksan, Gyeonggi-do, South Korea) for 10 min (see Figure S1 for the various concentrations used). The patterned F-SAM/PS brush guiding template was then obtained by dissolving the PS colloidal particle layer in toluene for 30 min.

### 2.3. Dewetting of the BCP Film on the Guiding Template

A 1.5 wt.% solution of polystyrene-block-polydimethylsiloxane (PS-*b*-PDMS) (Mw = 45.5 kg mol^−1^; PS (31 kg mol^−1^)-*b*−PDMS (14.5 kg mol^−1^), Polymer Source Inc.) in toluene or propylene glycol monomethyl ether acetate (PGMEA, 99.9 %, Samchun chemicals) was spin coated onto the prepatterned F-SAM/PS brush template to induce the dewetting of the BCP film. The BCP film was then solvent-vapor annealed by placing the sample in a glass chamber containing a 5:1 mixture of toluene and n-heptane (1.5 mL) for 5 h at room temperature. Afterward, the films were plasma etched (CF_4_, 5 s, 50 W; O_2_, 30 s, 90 W) to reveal the oxidized PDMS cylinder structures.

### 2.4. Imaging, Characterization, and Pattern Analyses

The surface morphologies of the BCP films and colloidal crystals were examined using scanning electron microscopy (SEM) (Sigma 500, Carl Zeiss, Oberkochen, Germany). The colloidal crystal samples were coated with Pt before SEM imaging. Contact angles were measured using a contact angle analyzer (phoenix 300 touch, SEO, Gyeonggi-do, South Korea). The number of rings (*n_r_*) in each sample was determined by examining 175 concentric ring patterns across 10 different SEM images.

## 3. Results

### 3.1. Fabrication of the Guiding Template

**Figure 1:**
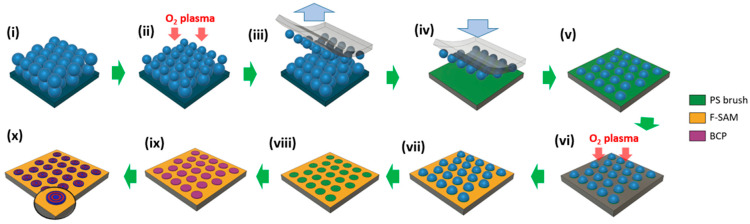
Schematic of the overall experimental process: (**i**) Multilayered colloidal crystal structure; (**ii**) O_2_ plasma etching of the surface layer of the colloidal crystal; (**iii**) peeling of the surface layer of the colloidal crystal using a PDMS stamp; (**iv**) transfer of the colloidal crystal monolayer to the PS brush substrate; (**v**) resulting non-close-packed hemispherical colloidal crystal monolayer on the PS brush substrate; (**vi**) O_2_ plasma etching to remove the exposed areas of the PS brush layer; (**vii**) F-SAM deposition on the exposed surfaces; (**viii**) F-SAM-patterned guiding template upon the removal of the colloidal particles; (**ix**) area-selective dewetting of the BCP thin film; (**x**) microphase separation of the BCP film via solvent vapor annealing.

Figure 1 shows the experimental process for the fabrication of dewetting-induced hierarchical patterns of block copolymers using a colloidal crystal template. Briefly, steps i–v illustrate the formation of a non-close-packed hemispherical colloidal crystal monolayer on a PS brush-coated substrate via thermal transfer [46]. The exposed PS brush layer is then removed by O_2_ plasma treatment (step vi); after etching, the PS brush area masked by the colloidal particles remains. Subsequently, the F-SAM HDFS is deposited on the etched areas of the substrate (step vii). A chemical-guiding template composed of a hexagonal array of circular PS brush features separated by an F-SAM layer is then obtained by dissolving the PS colloidal particles (step viii). A BCP solution is then spin coated onto the guiding template (step ix); the area-selective dewetting of the BCP thin film occurs by the local migration of the solution from the F-SAM-layered area to the PS brush-coated features. Finally, the dewetted BCP thin film is solvent annealed in a chamber containing a toluene-heptane (5:1) mixture to induce the self-assembly of PDMS cylinders in the PS matrix. CF_4_/O_2_ plasma etching then selectively removes the PDMS skin layer and PS domain to reveal the oxidized PDMS cylinders (step x).

**Figure 2:**
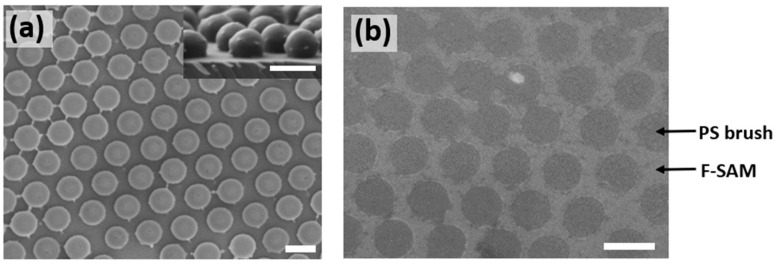
(**a**) SEM images of the vertical and lateral (inset) views of the transferred non-close-packed hemispherical colloidal crystal monolayer. (**b**) SEM images of the F-SAM/PS brush-patterned substrate after colloid removal. Darker and brighter regions represent the PS brush-coated and F-SAM-treated areas, respectively. All scale bars represent 400 nm.

An SEM image of the colloidal crystal monolayer transferred to a PS brush-coated silicon substrate is presented in Figure 2a. The non-close-packed features of the colloidal array enable sufficient separation between the masked PS brush-coated areas for the subsequent deposition of F-SAM. Moreover, the colloidal particles are deformed into a hemispherical shape during the transfer process because the heating of the substrate above the T_g_ of PS causes the partial melting of the PS colloidal particles, thereby flattening the contact area with the substrate (inset image of Figure 2a). This large contact area provides a sufficient masking area for the substrate during the O_2_ plasma etching and F-SAM deposition processes. Figure 2b presents an SEM image of the resulting F-SAM/PS brush-patterned substrate, showing a clear separation between the F-SAM-treated and PS brush-coated areas.

### 3.2. Dewetting of the BCP Film on the Guiding Template

**Figure 3:**
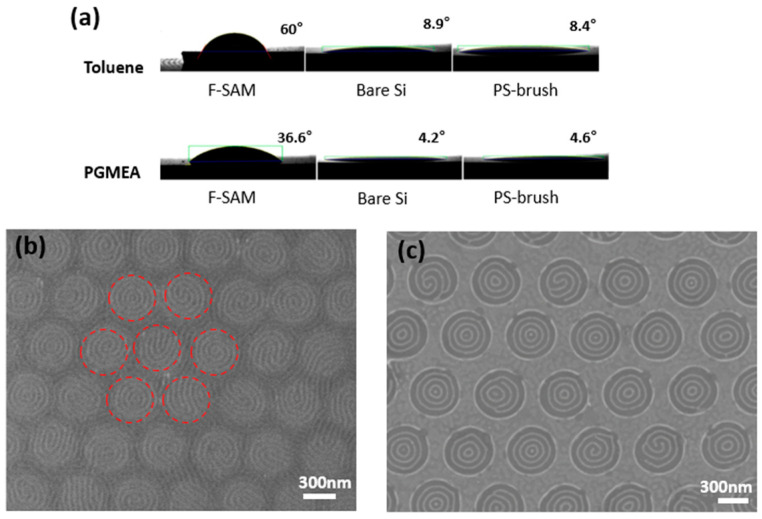
(**a**) Measured contact angles of toluene and PGMEA on various coated Si substrates. (**b**) SEM images of a dewetted BCP film on the guiding template after spin coating with BCP/PGMEA. Red circles indicate the PS brush-coated areas of the substrate. (**c**) SEM images of a dewetted BCP film on the guiding template after spin coating with BCP/toluene.

The choice of solvent at the dewetting stage is important because the dewetting of BCP occurs during the spin-coating process, where the local migration of the solution via solvent evaporation occurs. Toluene and PGMEA are the most widely used solvents for preparing PS-*b*-PDMS BCP solutions [47,48]. The contact angles of these two solvents on differently coated silicon substrates were investigated (Figure 3a). Regardless of the solvent used, the F-SAM-treated substrate shows the highest contact angles, which can be attributed to the extremely low surface energy imparted by the fluorinated interface [49]. Among the two solvents, toluene presents consistently higher contact angles than PGMEA on the various surfaces; this may be because the surface tension of toluene (28.5 mN/m) is relatively higher than that of PGMEA (26.9 mN/m) [50,51].

The driving force of the surface-directed dewetting process is the difference in wettability between the F-SAM-treated and PS brushed-coated surfaces (see Figure S2 for macroscopic dewetting studies of BCP films on F-SAM- and PS brush-coated surfaces). The contact angle experiment revealed that the difference in the wettability of toluene between these surfaces is larger than that of PGMEA; therefore, the dewetting phenomenon occurs to a higher extent when toluene is used as a solvent for the BCP solution. The experimental results (Figure 3b,c) are in good agreement with this expectation. When the BCP film is prepared via spin coating with BCP/PGMEA on the guiding template, dewetting is negligible (Figure 3b). Although a continuous film is formed without dewetting, the self-assembled pattern of the BCP film is affected by the chemical pattern underneath the film. As shown by the red circles in Figure 3b, the BCP film on the PS brush-coated areas forms a concentric or spiral-like pattern. The large surface energy difference between the F-SAM- and PS-coated areas induces chain mobility differences during the annealing process [52], which can affect pattern formation. This result is similar to that of the chemoepitaxy method which controls the pattern of the BCP film via a chemical pattern underneath the film [17,18,53]. Figure 3c shows an SEM image of a dewetted BCP film on the guiding template obtained by spin coating the BCP solution using toluene as the solvent. The BCP patterns are selectively formed on the PS brush-coated areas owing to the large difference in wettability between these and the F-SAM-treated substrate. The BCP patterns form concentric rings, similar to those formed on previously reported circular templates [54,55]. The concentration of the BCP solution also affects the final morphology of the dewetted film, as shown in Figure S3.

**Figure 4:**
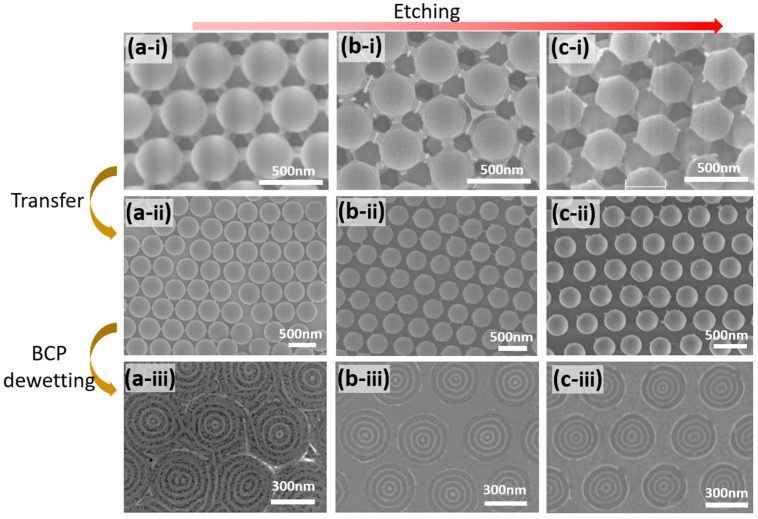
(**a-i**–**c-i**) Surface-etched multilayered colloidal crystals after O_2_ plasma etching times of (**a-i**) 2 min, (**b-i**) 3 min, and (**c-i**) 4 min. (**a-ii**–**c-ii**) Respective non-close-packed hemispherical colloidal crystal monolayers transferred from the surface-etched colloidal crystals shown in (**a-i**–**c-i**). (**a-iii**–**c-iii**) Dewetted BCP patterns formed on the guiding templates prepared from the colloidal crystal monolayers shown in (**a-ii**–**c-ii**).

An advantage of using a template formed from colloidal crystals is that the diameter and spacing of the final pattern can be adjusted by controlling the etching time or diameter of initial colloidal particles. Figure 4a-i–c-i shows the change in the particle size of the multilayered colloidal crystal surface layer before transfer, which depends on the O_2_ plasma etching time. The center-to-center interparticle distance remains constant throughout the etching process, whereas the particle diameter decreases and the interparticle spacing widens with increased etching time. Figure 4a-ii–c-ii shows SEM images of the hemispherical colloidal crystal monolayers after transfer from the respective multilayered crystals; here, the particle diameters and interparticle spacings are replicated after the transfer step. When the BCP is deposited and dewetted on the final templates, the resulting patterns also exhibit noticeable variations, as shown in Figure 4a-iii–c-iii.

When the initial colloidal crystal is etched for 2 min, the resulting BCP film pattern is poorly defined because of the narrow F-SAM area formed by the small interparticle spacing of the template (Figure 4a-iii). However, when the etching time is increased to 3 min, the resulting template provides sufficient space for the F-SAM deposition; consequently, the final BCP film exhibits clear feature separation after dewetting. The center-to-center distance of the 3 min-etched template is 430 nm, corresponding to the initial size of the colloidal particles and the average diameter of the final dewetted BCP film, which is 350 nm. The process affords a BCP pattern with four concentric rings (including the central dot), as shown in Figure 4b-iii. Furthermore, as the etching time is increased to 4 min, the average BCP pattern diameter decreased to 320 nm, forming a BCP pattern with three concentric rings (Figure 4c-iii).

### 3.3. Analysis of the BCP Concentric Ring Patterns

**Figure 5:**
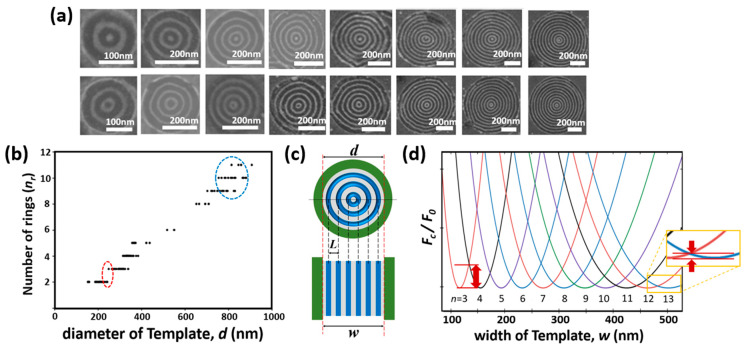
(**a**) SEM images of BCP concentric ring patterns fabricated with guiding templates of various sizes. Rows display the PDMS concentric rings with (top) and without (bottom) the PDMS central dot. (**b**) Number of concentric rings (*n_r_*) as a function of the diameter of the template (*d*). (**c**) Schematics of concentric rings formed in a circular trench template (top) and a line pattern formed in a linear trench template (bottom). (**d**) Free energy per confined polymer chain relative to the bulk free energy per polymer chain (*F_c_/F_0_*) as a function of the template width (*w*) for each value of *n*. Red arrows indicate the difference in the free energy between the commensurate and incommensurate states.

Figure 5a shows SEM images illustrating the morphology change of the BCP patterns depending on the size of the guiding template. Various diameter of F-SAM guiding templates (153–907 nm) were obtained by varying the etching time and the initial particle size of colloidal crystals. The number of concentric rings in each BCP pattern varies with the template size; however, differences in feature sizes within the same sample may occur because of the non-uniformity of the synthesized colloidal particles. To analyze the relationship between the feature dimensions and the template size, the number of rings (*n_r_*) was measured with respect to the template diameter (*d*) (Figure 5b). The concentric ring patterns inside the template may include either a PDMS dot or small PDMS ring in the center, as shown in the separated rows of Figure 5a. In the subsequent analysis, the central dot was categorized as a ring. The graph shows that as the diameter of the template (*d*) is increased, the number of concentric rings increases in a stepwise manner. This tendency can be explained by the difference in the free energy of the confined BCP molecules, which changes with the template diameter.

Turner’s free energy model has previously been used to explain the free energy changes of lamellae-forming BCP between two plates; this can also be applied to the formation of BCP cylinders in a confined template [56,57,58]. The concentric ring pattern differs from the line pattern associated with Turner’s free energy model; however, as shown in Figure 5c, the concentric ring pattern can be converted into a line pattern form that can be applied to this model, for example, BCP patterns with three concentric rings (*n_r_* = 3) formed in a circular template of diameter *d* can be converted into a line pattern form with a line number (*n*) of six in a template with a width *w*. The periodicity at the equilibrium state, *L*, is consistent in both the concentric ring and line patterns.

The simple equation form of Turner’s model provides an expression for the free energy per polymer chain under confinement (*F_c_*) relative to the free energy in the bulk polymer (*F_0_*) [57]:

(1) $$\frac{F_{c}}{F_{0}}=\frac{1}{3}\;\left(\lambda^{2}+\frac{2}{\lambda }\;\right)$$

where *λ* = *w*/*nL_0_*; *n* is the number of lamellae or cylinders; and *L_0_* is the natural periodicity of the BCP pattern. In this study, *L_0_* was measured as 38.5 nm.

Figure 5d shows the free energy as a function of *w* for each value of *n* calculated using Equation (1). This graph indicates that a specific *n* will be selected to yield the lowest free energy for a defined *w*. As *w* increases, the free energy penalty from the incommensurate state increases, leading to a transition from *n* to (*n +* 1). Consequently, as *w* increases, the value of *n* that yields the lowest free energy in the template also increases. This is in good agreement with the results shown in Figure 5b.

Notably, the free energy cost owing to the incommensurate state, as indicated by the red arrows in Figure 5d, decreases as *w* increases. This is because more BCP molecules share the tensile or compressive strain energy induced by the incommensurate state at a wider *w*; the incommensurate energy cost per molecule therefore becomes smaller. This phenomenon can also be observed in the experimental results shown in Figure 5b. In the transition region, the overlap in *n_r_* is small when *d* is small (as indicated by the red ellipse in Figure 5b); however, the overlaps become larger as *d* increases. For example, wide overlaps between *n_r_* values of 9 and11 were observed in the region of *d* = 800 nm (as indicated by the blue ellipse in Figure 5b).

### 3.4. Analysis of Common Defective Features in the BCP Patterns

**Figure 6:**
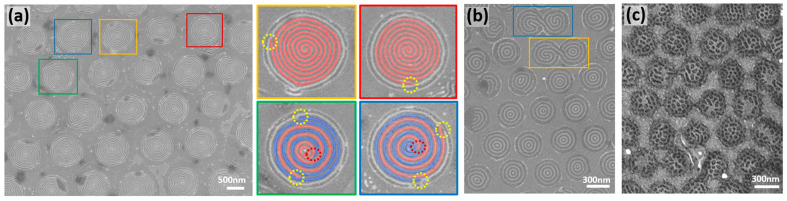
(**a**) SEM images of a dewetted BCP film on a guiding template prepared using 1100 nm colloidal particles. BCP patterns in the colored boxes exhibit spiral morphologies. Right-hand side panels show magnified images of the spiral structures (false-colorized), where the dotted circles indicate Y-junctions (yellow circles = diverging; red circles = converging). (**b**) Dewetted BCP film on a guiding template prepared using 430 nm colloidal particles. Colored boxes indicate merged BCP patterns from two adjacent template areas. (**c**) Dewetted BCP film with a perforated lamellar pattern.

In addition to the concentric ring pattern, defective features were formed on the circular guiding templates. The most common defect feature is the spiral structure, as shown in Figure 6a. In a previous study, we revealed that the spiral structures of a BCP film are created when the geometrical symmetry of the circular template is broken [55]. Therefore, BCP spiral structures could be fabricated by purposefully introducing artificial defects into a circular template [55,59].

The colored boxes in Figure 6a indicate spiral structures in a BCP pattern. Upon magnification, the images reveal that the spiral structures are accompanied by Y-junctions (as indicated by the yellow circles). In the common BCP line pattern, the Y-junction defects affect only adjacent areas (see Figure S4), whereas in the circular confined geometry, the presence of a Y-junction breaks the geometrical symmetry of the BCP pattern, leading to the formation of a spiral structure. In general, a BCP pattern is formed from the template edge and propagates inward [60,61]; therefore, it can be concluded that the inner spiral structure originates from the outer Y-junction. Here, the direction of the Y-junction determines the chirality of the spiral structure; a spiral arc is formed in the direction in which the Y-junction diverges. Y-junctions were therefore formed in different directions in the areas highlighted by the yellow and red boxes in Figure 6a, resulting in spirals with right- and left-handed chiralities, respectively. Moreover, the spiral structures in the green and blue boxes in Figure 6a indicate that the presence of two Y-junctions (yellow circles) affords a double spiral structure, where each Y-junction generates a separate spiral arc. In contrast, converging Y-junctions near the center of the patterned area (highlighted by the red circles) form in the direction opposite to that of the outer Y-junctions (yellow circles, diverging Y-junction), which results in the convergence of the two separate spiral arcs.

Another commonly observed defect is the merging of two adjacent templated patterns (Figure 6b). These double structured patterns form when the PDMS cylinders are connected between adjacent regions. Spiral-like structures are then generated owing to the geometrical asymmetry that arises as a result. Additionally, perforated lamellar patterns can be obtained by changing the solvent vapor annealing conditions (Figure 6c). This occurs because toluene and n-heptane can induce a selective swelling of the PS and PDMS domains, respectively (solubility parameters: δ_Tol_ = 18.2 MPa^1/2^, δ_Hep_ = 15.3 MPa^1/2^, δ_PS_ = 18.5 MPa^1/2^, and δ_PDMS_ = 15.5 MPa^1/2^) [62]. For example, increasing the n-heptane content of the solvent mixture (resulting in a 1:1 ratio) at the annealing step increases the effective volume fraction of the PDMS domain, thereby forming the perforated lamellar morphology shown in Figure 6c [47].

## 4. Conclusions

In this study, we demonstrated the formation of dewetting-induced hierarchical patterns using two self-assembled materials: BCPs and colloidal crystals. The combination of these two self-assembly methods successfully generates multiscale hierarchical patterns because the length scales afforded by the periodic colloidal crystal structures enables the formation of distinct F-SAM- and PS brush-coated regions that are suitable for guiding the BCP patterns. The use of a dewetting method directed by such a chemical template results in a simple and versatile patterning process that does not require a topographic template. The dewetted BCP films exhibit concentric ring patterns with internal structures that can be controlled by the diameter or spacing of the fabricated circular guiding template. A free energy model was applied to analyze these patterns; it was subsequently found that the interaction of the relative free energy of the polymer chains with respect to the template width determines the number of concentric rings formed in the final pattern. In addition, the formation of common spiral defects was investigated; the results revealed that the formation of Y-junction defects was closely related to the generation of spiral morphologies in the BCP films.

This hierarchical patterning method is of particular interest because it provides a link between the nanoscale patterning offered by BCPs and the long-range microscale or sub-microscale patterning afforded by colloidal crystal templates. The combined techniques described here therefore offer considerable potential for the development of micro/nanofabrication methods for patterning diverse functional materials.

## Data Availability

The data presented in this study are available on request from the corresponding author.

## Supplementary Materials

The following supporting information can be downloaded at https://www.mdpi.com/article/10.3390/polym15040897/s1: Figure S1: SEM images of dewetted block copolymer (BCP) films formed on F-SAM/PS brush-patterned substrates. F-SAM layer was prepared using various concentrations of heptadefluoro-1,1,2,2-tetrahydrodecyl trichlorosilane (HDFS)—(a) 0.1 wt.%, (b) 0.2 wt.%, and (c) 0.5 wt.%; Figure S2: Optical microscopy images of BCP films on (a) PS brush-coated and (b) F-SAM-coated substrates. Toluene was used as the solvent for the BCP solution; Figure S3: SEM images of BCP films spin coated with 2.0 wt.% BCP solutions in (a) PGMEA and (b) toluene; Figure S4: SEM images of BCP films spin coated with 2.0 wt.% BCP solutions in (a) PGMEA and (b) toluene; Figure S4: SEM images of Y-junctions (yellow circles) in the BCP* pattern (parallel cylinders) formed without the use of a template.

## References

[1] Herr D.J.C. (2011). Directed Block Copolymer Self-Assembly for Nanoelectronics Fabrication. J. Mater. Res..

[2] Kim H.-C., Park S.-M., Hinsberg W.D. (2010). Block Copolymer Based Nanostructures: Materials, Processes, and Applications to Electronics. Chem. Rev..

[3] Ditte K., Perez J., Chae S., Hambsch M., Al-Hussein M., Komber H., Formanek P., Mannsfeld S.C.B., Fery A., Kiriy A. (2020). Ultrasoft and High-Mobility Block Copolymers for Skin-Compatible Electronics. Adv. Mater..

[4] Yang Y., Kim H., Xu J., Hwang M.-S., Tian D., Wang K., Zhang L., Liao Y., Park H.-G., Yi G.-R. (2018). Responsive Block Copolymer Photonic Microspheres. Adv. Mater..

[5] Poutanen M., Guidetti G., Gröschel T.I., Borisov O.V., Vignolini S., Ikkala O., Gröschel A.H. (2018). Block Copolymer Micelles for Photonic Fluids and Crystals. ACS Nano.

[6] Song D.-P., Li C., Li W., Watkins J.J. (2016). Block Copolymer Nanocomposites with High Refractive Index Contrast for One-Step Photonics. ACS Nano.

[7] Park T.H., Eoh H., Jung Y., Lee G.-W., Lee C.E., Kang H.S., Lee J., Kim K.-B., Ryu D.Y., Yu S. (2020). Thermo-Adaptive Block Copolymer Structural Color Electronics. Adv. Funct. Mater..

[8] Hägglund C., Zeltzer G., Ruiz R., Thomann I., Lee H.-B.-R., Brongersma M.L., Bent S.F. (2013). Self-Assembly Based Plasmonic Arrays Tuned by Atomic Layer Deposition for Extreme Visible Light Absorption. Nano Lett..

[9] Cha S.K., Mun J.H., Chang T., Kim S.Y., Kim J.Y., Jin H.M., Lee J.Y., Shin J., Kim K.H., Kim S.O. (2015). Au-Ag Core-Shell Nanoparticle Array by Block Copolymer Lithography for Synergistic Broadband Plasmonic Properties. ACS Nano.

[10] Nunes S.P., Car A. (2013). From Charge-Mosaic to Micelle Self-Assembly: Block Copolymer Membranes in the Last 40 Years. Ind. Eng. Chem. Res..

[11] Uehara H., Kakiage M., Sekiya M., Sakuma D., Yamonobe T., Takano N., Barraud A., Meurville E., Ryser P. (2009). Size-Selective Diffusion in Nanoporous but Flexible Membranes for Glucose Sensors. ACS Nano.

[12] Luo Y., Wang X., Zhang R., Singh M., Ammar A., Cousins D., Hassan M.K., Ponnamma D., Adham S., Al-Maadeed M.A.A. (2020). Vertically Oriented Nanoporous Block Copolymer Membranes for Oil/Water Separation and Filtration. Soft Matter.

[13] Young W.-S., Kuan W.-F., Epps T.H. (2013). Block Copolymer Electrolytes for Rechargeable Lithium Batteries. J. Polym. Sci. B Polym. Phys..

[14] Jeong C.K., Baek K.M., Niu S., Nam T.W., Hur Y.H., Park D.Y., Hwang G.-T., Byun M., Wang Z.L., Jung Y.S. (2014). Topographically-Designed Triboelectric Nanogenerator via Block Copolymer Self-Assembly. Nano Lett..

[15] Yang H., Shi X., Chu S., Shao Z., Wang Y. (2021). Design of Block-Copolymer Nanoporous Membranes for Robust and Safer Lithium-Ion Battery Separators. Adv. Sci..

[16] Lin Z., Guo X., Yang Y., Tang M., Wei Q., Yu H. (2021). Block Copolymer Electrolyte with Adjustable Functional Units for Solid Polymer Lithium Metal Battery. J. Energy Chem..

[17] Kim S.O., Solak H.H., Stoykovich M.P., Ferrier N.J., De Pablo J.J., Nealey P.F. (2003). Epitaxial Self-Assembly of Block Copolymers on Lithographically Defined Nanopatterned Substrates. Nature.

[18] Stoykovich M.P., Müller M., Kim S.O., Solak H.H., Edwards E.W., De Pablo J.J., Nealey P.F. (2005). Directed Assembly of Block Copolymer Blends into Nonregular Device-Oriented Structures. Science.

[19] Bita I., Yang J.K.W., Jung Y.S., Ross C.A., Thomas E.L., Berggren K.K. (2008). Graphoepitaxy of Self-Assembled Block Copolymers on Two-Dimensional Periodic Patterned Templates. Science.

[20] Yang J.K.W., Jung Y.S., Chang J.-B., Mickiewicz R.A., Alexander-Katz A., Ross C.A., Berggren K.K. (2010). Complex Self-Assembled Patterns Using Sparse Commensurate Templates with Locally Varying Motifs. Nat. Nanotechnol..

[21] Jeong S.-J., Kim J.Y., Kim B.H., Moon H.-S., Kim S.O. (2013). Directed Self-Assembly of Block Copolymers for Next Generation Nanolithography. Mater. Today.

[22] Zhang J., Li Y., Zhang X., Yang B. (2010). Colloidal Self-Assembly Meets Nanofabrication: From Two-Dimensional Colloidal Crystals to Nanostructure Arrays. Adv. Mater..

[23] Jeong U., Wang Y., Ibisate M., Xia Y. (2005). Some New Developments in the Synthesis, Functionalization, and Utilization of Monodisperse Colloidal Spheres. Adv. Funct. Mater..

[24] Ke Y., Ye S., Hu P., Jiang H., Wang S., Yang B., Zhang J., Long Y. (2019). Unpacking the Toolbox of Two-Dimensional Nanostructures Derived from Nanosphere Templates. Mater. Horiz..

[25] Oliveira R.D., Mouquinho A., Centeno P., Alexandre M., Haque S., Martins R., Fortunato E., Águas H., Mendes M.J. (2021). Colloidal Lithography for Photovoltaics: An Attractive Route for Light Management. Nanomaterials.

[26] Qiu T., Akinoglu E.M., Luo B., Konarova M., Yun J.-H., Gentle I.R., Wang L. (2022). Nanosphere Lithography: A Versatile Approach to Develop Transparent Conductive Films for Optoelectronic Applications. Adv. Mater..

[27] Li J., Hu Y., Yu L., Li L., Ji D., Li L., Hu W., Fuchs H. (2021). Recent Advances of Nanospheres Lithography in Organic Electronics. Small.

[28] Brassat K., Kool D., Bürger J., Lindner J.K.N. (2018). Hierarchical Nanopores Formed by Block Copolymer Lithography on the Surfaces of Different Materials Pre-Patterned by Nanosphere Lithography. Nanoscale.

[29] Baek K.M., Kim J.M., Jeong J.W., Lee S.Y., Jung Y.S. (2015). Sequentially Self-Assembled Rings-in-Mesh Nanoplasmonic Arrays for Surface-Enhanced Raman Spectroscopy. Chem. Mater..

[30] Brassat K., Kool D., Nallet C.G.A., Lindner J.K.N. (2019). Understanding Film Thickness-Dependent Block Copolymer Self-Assembly by Controlled Polymer Dewetting on Prepatterned Surfaces. Adv. Mater. Interfaces.

[31] Das A., Mukherjee R. (2021). Feature Size Modulation in Dewetting of Nanoparticle-Containing Ultrathin Polymer Films. Macromolecules.

[32] Huang J., Kim F., Tao A.R., Connor S., Yang P. (2005). Spontaneous Formation of Nanoparticle Stripe Patterns through Dewetting. Nat. Mater..

[33] Sehgal A., Ferreiro V., Douglas J.F., Amis E.J., Karim A. (2002). Pattern-Directed Dewetting of Ultrathin Polymer Films. Langmuir.

[34] Giermann A.L., Thompson C.V. (2005). Solid-State Dewetting for Ordered Arrays of Crystallographically Oriented Metal Particles. Appl. Phys. Lett..

[35] Bhandaru N., Mukherjee R. (2021). Ordering in Dewetting of a Thin Polymer Bilayer with a Topographically Patterned Interface. Macromolecules.

[36] Choi S.Y., Lee C., Lee J.W., Park C., Kim S.H. (2012). Dewetting-Induced Hierarchical Patterns in Block Copolymer Films. Macromolecules.

[37] Brassat K., Lindner J.K.N. (2019). Nanoscale Block Copolymer Self-Assembly and Microscale Polymer Film Dewetting: Progress in Understanding the Role of Interfacial Energies in the Formation of Hierarchical Nanostructures. Adv. Mater. Interfaces.

[38] Farrell R.A., Kehagias N., Shaw M.T., Reboud V., Zelsmann M., Holmes J.D., Torres C.M.S., Morris M.A. (2011). Surface-Directed Dewetting of a Block Copolymer for Fabricating Highly Uniform Nanostructured Microdroplets and Concentric Nanorings. ACS Nano.

[39] Lee Y.-C., Sun Y.-S., Liou J.-Y., Chuang W.-T. (2012). Hierarchically-Responded Assembly of Block Copolymer Thin Films with Stimuli of Varied Solvent Selectivity. Polymer.

[40] Lee J.H., Choi H.-J., Lee C., Song S.W., Lee J.B., Huh D., Nam Y.S., Jeon D.Y., Lee H., Jung Y.S. (2018). Spontaneous Registration of Sub-10 nm Features Based on SubZero Celsius Spin-Casting of Self-Assembling Building Blocks Directed by Chemically Encoded Surfaces. ACS Nano.

[41] Kim T.H., Hwang J., Hwang W.S., Huh J., Kim H.-C., Kim S.H., Hong J.M., Thomas E.L., Park C. (2008). Hierarchical Ordering of Block Copolymer Nanostructures by Solvent Annealing Combined with Controlled Dewetting. Adv. Mater..

[42] Kim T.-H., Hwang J., Acharya H., Park C. (2010). Ordered Nanostructure of PS-b-PEO Copolymer by Solvent Annealing with Mixture of Benzene/Water Vapor and Its Micropattern Fabrication. J. Nanosci. Nanotechnol..

[43] Kim J.H., Chainey M., El-Aasser M.S., Vanderhoff J.W. (1992). Emulsifier-Free Emulsion Copolymerization of Styrene and Sodium Styrene Sulfonate. J. Polym. Sci. A Polym. Chem..

[44] Ha S.T., Park O.O., Im S.H. (2010). Size Control of Highly Monodisperse Polystyrene Particles by Modified Dispersion Polymerization. Macromol. Res..

[45] Choi H.K., Kim M.H., Im S.H., Park O.O. (2009). Fabrication of Ordered Nanostructured Arrays Using Poly(Dimethylsiloxane) Replica Molds Based on Three-Dimensional Colloidal Crystals. Adv. Funct. Mater..

[46] Choi H.K., Yang Y.J., Park O.O. (2014). Hemispherical Arrays of Colloidal Crystals Fabricated by Transfer Printing. Langmuir.

[47] Jung Y.S., Ross C.A. (2009). Solvent-Vapor-Induced Tunability of Self-Assembled Block Copolymer Patterns. Adv. Mater..

[48] Son J.G., Hannon A.F., Gotrik K.W., Alexander-Katz A., Ross C.A. (2010). Hierarchical Nanostructures by Sequential Self-Assembly of Styrene-Dimethylsiloxane Block Copolymers of Different Periods. Adv. Mater..

[49] Barriet D., Lee T.R. (2003). Fluorinated Self-Assembled Monolayers: Composition, Structure and Interfacial Properties. Curr. Opin. Colloid Interface Sci..

[50] Jasper J.J. (1972). The Surface Tension of Pure Liquid Compounds. J. Phys. Chem. Ref. Data.

[51] Yang P.-C., Ting Y.-X., Gu S., Gandomi Y.A., Li J., Hsieh C.-T. (2021). Effect of Solvent on Fluorescence Emission from Polyethylene Glycol-Coated Graphene Quantum Dots under Blue Light Illumination. Nanomaterials.

[52] Jung Y.S., Ross C.A. (2009). Well-Ordered Thin-Film Nanopore Arrays Formed Using a Block-Copolymer Template. Small.

[53] Ruiz R., Kang H., Detcheverry F., Dobisz E., Kercher D.S., Albrecht T.R., De Pablo J.J., Nealey P.F. (2008). Density Multiplication and Improved Lithography by Directed Block Copolymer Assembly. Science.

[54] Jung Y.S., Jung W., Ross C.A. (2008). Nanofabricated Concentric Ring Structures by Templated Self-Assembly of a Diblock Copolymer. Nano Lett..

[55] Choi H.K., Chang J.-B., Hannon A.F., Yang J.K.W., Berggren K.K., Alexander-Katz A., Ross C.A. (2017). Nanoscale Spirals by Directed Self-Assembly. Nano Futures.

[56] Turner M.S. (1992). Equilibrium Properties of a Diblock Copolymer Lamellar Phase Confined between Flat Plates. Phys. Rev. Lett..

[57] Yun H.S., Do H.W., Berggren K.K., Ross C.A., Choi H.K. (2020). Commensurability-Driven Orientation Control during Block Copolymer Directed Self-Assembly. ACS Appl. Mater. Interfaces.

[58] Xu J., Russell T.P., Checco A. (2012). Lattice Deformation and Domain Distortion in the Self-Assembly of Block Copolymer Thin Films on Chemical Patterns. Small.

[59] Lee G.H., Kim S., Kim Y., Jang M.S., Jung Y.S. (2020). Simulation and Fabrication of Nanoscale Spirals Based on Dual-Scale Self-Assemblies. ACS Appl. Mater. Interfaces.

[60] Jeong J.W., Park W.I., Kim M.-J., Ross C.A., Jung Y.S. (2011). Highly Tunable Self-Assembled Nanostructures from a Poly(2-vinylpyridine-b-dimethylsiloxane) Block Copolymer. Nano Lett..

[61] Black C.T., Bezencenet O. (2004). Nanometer-Scale Pattern Registration and Alignment by Directed Diblock Copolymer Self-Assembly. IEEE Trans. Nanotechnol..

[62] Barton A.F. (1991). CRC Handbook of Solubility Parameters and Other Cohesion Parameters.

