# Supplementary material for: Dewetting-Induced Hierarchical Self-Assembly of Block Copolymers Templated by Colloidal Crystals
Source: Polymers (Basel). 2023 Feb 11;15(4):897. doi: 10.3390/polym15040897 (PMC9961777; doi:10.3390/polym15040897)
Supplement: Supplementary file 1 [file polymers-15-00897-s001.zip › polymers-2202778-supplementary.pdf]

# Supporting information

## Dewetting-induced hierarchical self-assembly of block copolymers templated by colloidal crystals

Dong Hwan Kim, Hong Gu Kwon, Hong Kyoon Choi**

*Division of Advanced Materials Engineering, Kongju National University, 1223-24Cheonan-daero, Seobuk-gu, Cheonan-si, Chungnam, 31080, South Korea.*

*Corresponding author

hkchio@kongju.ac.kr

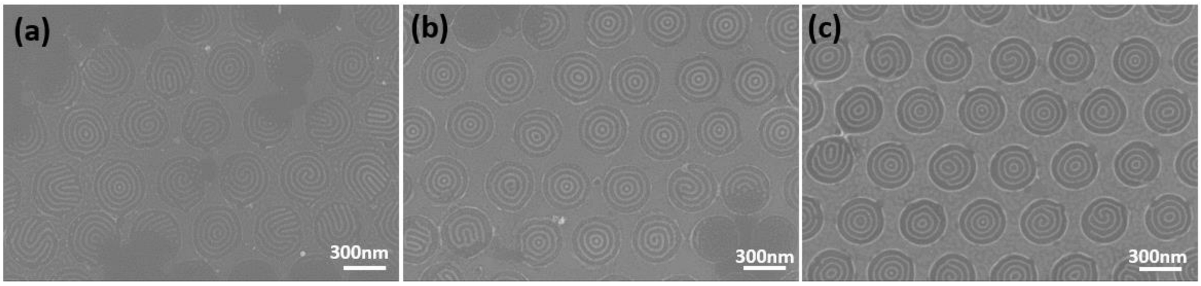


**Figure S1**. SEM images of dewetted block copolymer (BCP) films formed on F-SAM/PS brush-patterned substrates. F-SAM layer was prepared using various concentrations of heptadefluoro-1,1,2,2-tetrahydrodecyl trichlorosilane (HDFS) - (a) 0.1 wt.%, (b) 0.2 wt.%, and (c) 0.5 wt.%

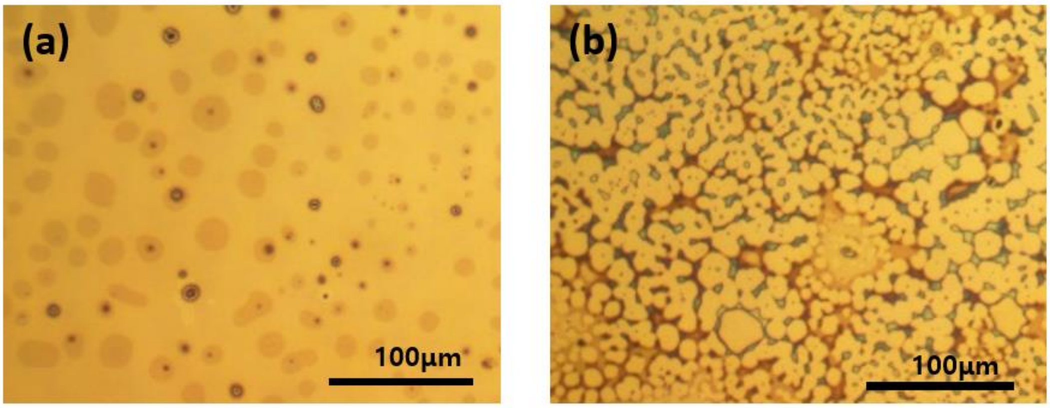


**Figure S2**. Optical microscopy images of BCP* film on (a) PS-brush coated substrate and (b) F-SAM deposited substrate. Toluene was used as the solvent for the BCP solution.

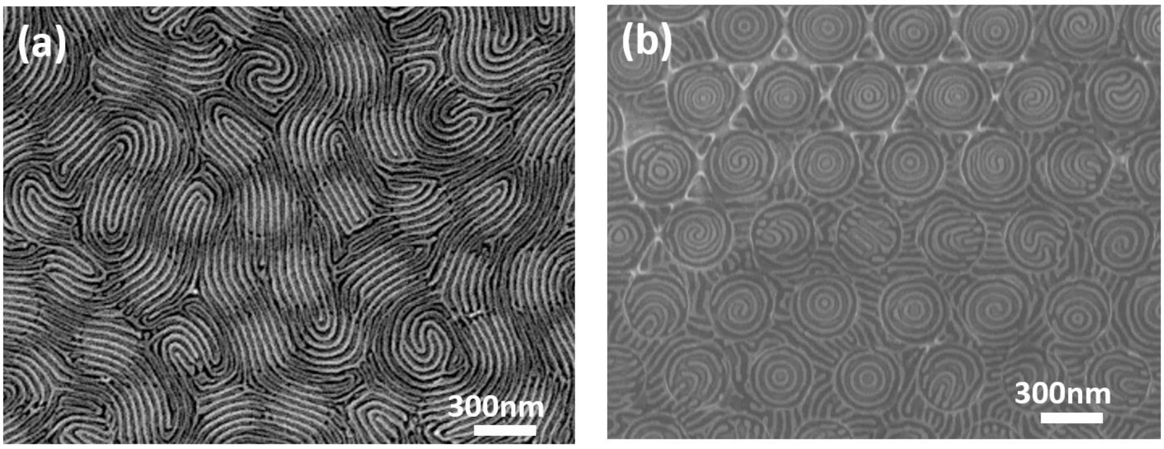


**Figure S3**. SEM images of BCP films spin-coated with 2.0 wt.% BCP solutions in (a) PGMEA and (b) toluene. (For comparison, see Figure 3(b), (c) with 1.5 wt.% BCP solutions)

In both samples, a bilayer of PDMS cylinders was formed as the film thickness increased. For the 2.0 wt.% BCP/toluene solution (Figure S3(b)), dewetting did not occur, and the BCP film remained in the F-SAM treated area, in contrast to that observed in the 1.5 wt.% BCP/toluene solution (Figure 3(c)).

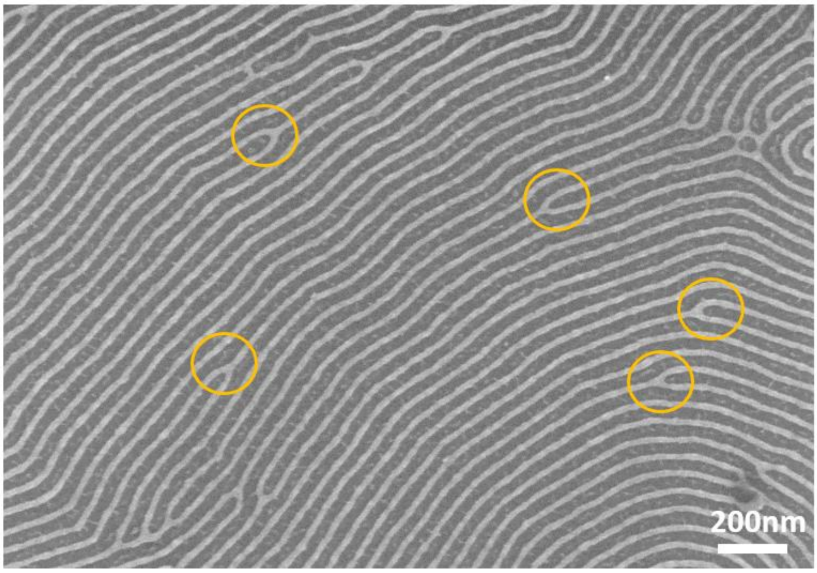


**Figure S4**. SEM images of Y-junctions (yellow circles) in the BCP* pattern (parallel cylinders) formed without the use of a template.

** PS-b-PDMS (Mw: 45.5 kg/mol, PS (31 kg/mol)-b−PDMS (14.5 kg/mol, SD45)*
